# High-Performance Hydrogen Sensing at Room Temperature via Nb-Doped Titanium Oxide Thin Films Fabricated by Micro-Arc Oxidation

**DOI:** 10.3390/nano15020124

**Published:** 2025-01-16

**Authors:** Chilou Zhou, Zhiqiu Ye, Yue Tan, Zhenghua Wu, Xinyi Guo, Yinglin Bai, Xuying Xie, Zilong Wu, Ji’an Feng, Yao Xu, Bo Deng, Hao Wu

**Affiliations:** 1 School of Mechanical and Automobile Engineering, South China University of Technology, Guangzhou 510641, China; mezcl@scut.edu.cn (C.Z.); yeyeah2001@163.com (Z.Y.); mezlwu@outlook.com (Z.W.); 2 Guangdong Institute of Special Equipment Inspection and Research, Foshan 510655, China; 19566831350@163.com (Y.X.); dengbo@gdsei.org.cn (B.D.); 3 Guangdong Key Laboratory of Materials and Equipment in Harsh Marine Environment, School of Ocean Engineering, Guangzhou Maritime University, Guangzhou 510725, China; wzh13423945193@163.com (Z.W.); xinyiguo089@163.com (X.G.); maxbaulfield77@outlook.com (Y.B.); xiexuying060330@163.com (X.X.); jafeng039@hotmail.com (J.F.)

**Keywords:** micro-arc oxidation, hydrogen sensor, Nb-doped titanium oxide, semiconductor

## Abstract

Metal oxide semiconductor (MOS) hydrogen sensors offer advantages, such as high sensitivity and fast response, but their challenges remain in achieving low-cost fabrication and stable operation at room temperature. This study investigates Nb-doped TiO_2_ (NTO) thin films prepared via a one-step micro-arc oxidation (MAO) with the addition of Nb_2_O_5_ nanoparticles into the electrolyte for room-temperature hydrogen sensing. The characterization results revealed that the incorporation of Nb_2_O_5_ altered the film’s morphology and phase composition, increasing the Nb content and forming a homogeneous composite thin film. Hydrogen sensing tests demonstrated that the NTO samples exhibited significantly improved sensitivity, selectivity, and stability compared to undoped TiO_2_. Among the fabricated samples, NTO thin film prepared at Nb_2_O_5_ concentration of 6 g/L (NTO-6) showed the best performance, with a broad detection range, excellent sensitivity, rapid response, and good specificity to hydrogen. A strong linear relationship between response values and hydrogen concentration (10–1000 ppm) highlights its potential for precise hydrogen detection. The enhanced hydrogen sensing mechanism of NTO thin films primarily stems from the influence of Nb_2_O_5_; nanoparticles doping in the anatase-phase TiO_2_ structure on the semiconductor surface depletion layer, as well as the improved charge transfer and additional adsorption sites provided by the Nb/Ti composite metal oxides, such as TiNb_2_O_7_ and Ti_0.95_Nb_0.95_O_4_. This study demonstrates the potential of MAO-fabricated Nb-doped TiO_2_ thin films as efficient and reliable hydrogen sensors operating at room temperature, offering a pathway for novel gas-sensing technologies to support clean energy applications.

## 1. Introduction

Hydrogen has gained substantial attention as a clean and sustainable energy source, offering an alternative to fossil fuels that can help mitigate environmental challenges, such as greenhouse gas emissions and global warming [1,2,3]. Its utilization, however, requires significant advancements in sensing technologies to ensure safe production, storage, and usage due to its flammability and the fact that it is odorless, colorless, and can easily leak [4]. Hence, the development of reliable hydrogen sensors that are capable of detecting hydrogen at room temperature with high sensitivity, selectivity, and a fast response remains a priority in both industrial and research domains. Among different types of hydrogen sensors, metal oxide semiconductors (MOSs) have garnered significant interest due to their ability to detect a wide range of gasses at low concentrations [5,6]. Titanium oxide (TiO_2_), in particular, is well known for its chemical stability, low cost, and versatility in gas sensing applications [7]. However, pristine TiO_2_ thin films often face limitations in terms of sensitivity and operational temperature, making them less suitable for practical applications that require room-temperature detection [7,8]. To overcome these limitations, various strategies have been explored, including surface modifications and doping. In this context, doping titanium oxide with transition metals, such as zinc [9], tin [10], silver [11], aluminum [12], copper [13], and niobium has emerged as a promising approach for enhancing the gas-sensing properties by altering its electrical and surface characteristics.

Niobium (Nb) doping is particularly attractive because of its unique influence on the electronic structure of TiO_2_, effectively increasing the concentration of free carriers and improving electrical conductivity. Nb-doped titanium oxide (NTO) has been investigated extensively in recent years for applications in photocatalysis [14], solar cells [15], lithium batteries [16], and gas sensors [17,18,19,20,21] due to its modified electronic properties and improved carrier mobility. Notably, most Nb-doped TiO_2_ sensors developed are composed of TiO_2_ powder and are limited to sensing at elevated temperatures, typically above 150 °C [21]. Li et al. [22] developed a capacitor-like sensor with a Pt/Nb-TiO_2_/Pt structure for hydrogen detection at 100 °C. However, unexpectedly, the Nb-doped TiO_2_ sensor exhibited a lower response compared to the undoped Pt/TiO_2_/Pt sensor due to lower surface roughness and porosity. Liu et al. [17] fabricated Nb-doped anatase-type titania nanotubes through anodization of Ti35Nb alloy and further annealing, achieving hydrogen sensitivity ranging from 50 ppm to 2% at room temperature. However, the observed response change, ranging narrowly from 7.7% to 41%, is insufficient for precise H_2_ concentration measurements. Bao et al. [21] synthesized through a process combining seed layer deposition, hydrothermal treatment, and subsequent annealing. The resulting NTO thin film exhibits an Nb-doped rutile/anatase TiO_2_ heterophase junction structure, which demonstrates an enhanced hydrogen response and an expanded detection range compared to non-doped TiO_2_. Moreover, the precise role of Nb doping in enhancing the hydrogen sensing properties of TiO_2_ thin films is still a subject of ongoing research, and more studies are required to develop an optimized NTO-based hydrogen-sensitive material that can operate effectively at room temperature.

Micro-arc oxidation (MAO) is an electrochemical technique that has been widely employed for producing porous oxide coatings on metals such as titanium [23]. It offers several advantages, including simplicity, cost-effectiveness, and the ability to fabricate films with controllable content and thickness. The MAO process involves the application of a high voltage to a metal substrate immersed in an electrolyte, resulting in the formation of an oxide film with a unique micro-porous structure [24]. This porous structure provides a large specific surface area, which is advantageous for gas adsorption and, consequently, for sensing applications. Recent research has demonstrated the potential of MAO for synthesizing doped oxide films [25,26,27]. Hence, it is possible to form a doped oxide thin film by MAO in one step, which can significantly reduce fabrication costs and improve process efficiency and scalability.

In this work, we attempt to realize this idea by systematically studying the hydrogen sensing properties of Nb-doped TiO_2_ thin films prepared by one-step micro-arc oxidation. The key objectives of this study are to elucidate the relationship between the MAO processing conditions and the resulting film characteristics, and to determine how these characteristics influence the hydrogen sensing behavior. We employ a combination of structural, morphological, and electrical characterization techniques to provide insights into the factors that govern the gas-sensing response of NTO thin films.

## 2. Materials and Methods

### 2.1. The Fabrication of Nb Doped TiO_2_ (NTO) Thin Film

**Figure 1:**
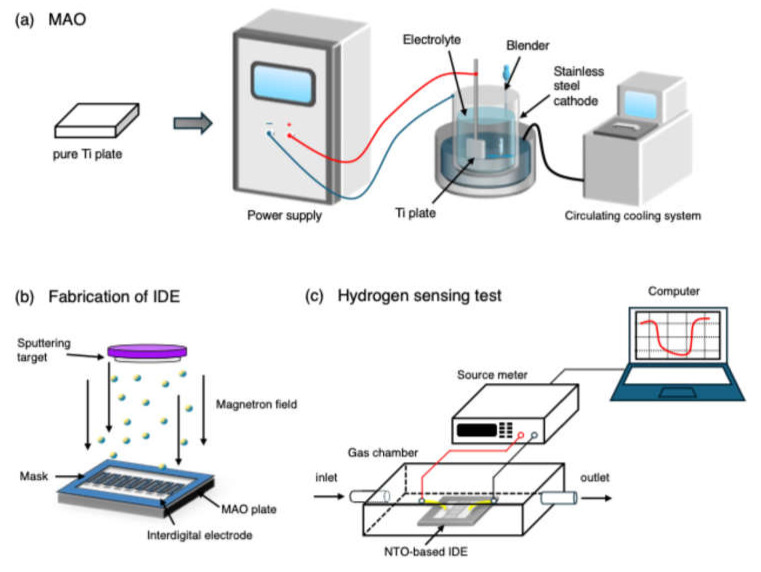
Schematic illustrations depicting (**a**) the MAO process, (**b**) magnetron sputtering for interdigital electrode (IDE) fabrication, and (**c**) the hydrogen sensing test.

Nb-doped TiO_2_ was synthesized in a single step using micro-arc oxidation (MAO). The 99.99% pure titanium plates (Wenghe Metal Materials Co., Ltd., Hefei, China) were cut into 25 × 25 × 2 mm^3^, ground with various grits of sandpaper, and used as substrate. As shown in Figure 1a, the MAO process was conducted using a custom-built system equipped with a DC pulsed power supply (Plasma Technology Ltd., Hong Kong SAR, China) and a stainless-steel container serving as the cathode. A cooling system and a mechanical stirrer were employed to maintain the electrolyte temperature below 50 °C. The electrolyte was prepared by dissolving 7 g/L of Na_3_PO_4_ and 1 g/L NaOH in deionized water with thorough stirring. The substrate was subjected to MAO treatment in constant current mode. The MAO process parameters were as follows: current density of 7.5 A/dm^2^, stirring speed of 300 rpm, frequency of 400 Hz, duty cycle of 30%, and treatment duration of 10 minutes. To introduce Nb doping, different amounts of niobium pentoxide (Nb_2_O_5_) nanoparticles with the average size of 20 nm were added to the electrolyte. After the experiment, the sample was quickly rinsed under the faucet, then ultrasonically cleaned in deionized water and anhydrous ethanol. The samples prepared in electrolytes with Nb_2_O_5_ concentrations of 0, 2, 4, 6, 8, and 10 g/L were marked as TO, NTO-2, NTO-4, NTO-6, NTO-8, and NTO-10, respectively.

### 2.2. The Fabrication of Hydrogen Sensor

As shown in Figure 1b, the sensor electrode was prepared by magnetron sputtering deposition interdigitated electrode (IDE) on the as-prepared MAO sample. Firstly, a stainless-steel mask with an IDE pattern was placed on the MAO sample. Then, the samples were placed in a magnetron sputterer (CCU-010, Safematic, Zizers, Switzerland) for a 300 s vacuum coating at a chamber base pressure below 5 × 10^−5^ Pa. A Pt interfinger electrode with 500 μm fingers and a 500 μm electrode gap was then formed.

### 2.3. Characterization

The fabricated NTO thin film was characterized by X-ray diffraction (XRD, Rigaku SmartLab, Tokyo, Japan) using Cu-Kα radiation at a scanning speed of 10°/min to determine its crystal structure. A scanning electron microscope (SEM, Zeiss Crossbeam 350, Oberkochen, Germany) coupled with energy-dispersive X-ray spectroscopy (EDS, Oxford instrument, Abingdon, UK) was employed to determine the film’s morphology and the elemental composition of the sample surface and cross-section. X-ray photoelectron spectroscopy (XPS, Kratos AXIS SUPRA+, Shimadzu Co., Kyoto, Japan) was used to study the chemical states of the synthetic material.

### 2.4. Measurement of the Gas Sensor

The gas response of the fabricated sensors was tested in a self-developed sensing performance device at room temperature (25 °C) (Figure 1c). This device consists of a stainless-steel gas chamber for the samples’ reaction with hydrogen. The hydrogen sensing tests were conducted by continuously introducing a hydrogen/nitrogen gas mixture with a specific concentration into the chamber, followed by dry air, to enable the sensor’s recovery. Two mass flow controllers (MFCs) were employed to precisely regulate the flow rates of hydrogen and nitrogen, ensuring accurate control of hydrogen concentration, while a source meter instrument (Keithley 2450, Tektronix Inc., Beaverton, OR, USA) was used to measure the resistance of the samples. The sensor response (S) is defined as the ratio of the resistance change to the baseline resistance in air, which can be calculated by the formula below [28]:

(1) $$S=\frac{R_{a}-R_{g}}{R_{a}}\times 100\%$$

where $R_{a}$ and $R_{g}$ represent the resistance in dry air and in the testing gas hydrogen, respectively. The $t_{90}$ is the time when the resistance reaches 90% of ($R_{a}-R_{g}$), $t_{0}$ is the time when the resistance is $R_{a}$. The response time $t_{res}$ is defined as the time it takes for the sensor to reach 90% of the resistance change from $R_{a}$ to $R_{g}$. Similarly, the recovery time $t_{rec}$ is the time required for the sensor to return to 90% of its initial resistance $R_{a}$. The selectivity of the sensor was tested by using 1000 ppm concentrations of carbon monoxide (CO), ammonia (NH_3_), and methane (CH_4_), each mixed with nitrogen, and the results were compared to 1000 ppm of H_2_.

## 3. Results

### 3.1. Voltage–Time Curves of MAO

**Figure 2:**
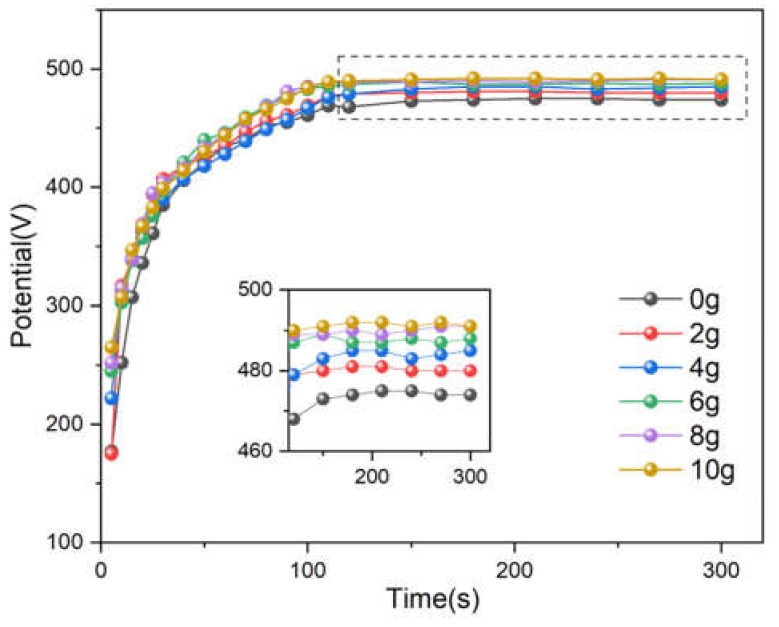
The voltage-time curves of MAO process in electrolytes containing varying concentrations (0–10 g/L) of Nb_2_O_5_ nanoparticles.

Figure 2 illustrates the relationship between the MAO reaction time and voltage under constant current mode in electrolytes containing varying concentrations (0–10 g/L) of Nb_2_O_5_ nanoparticles. The voltage–time curves of different samples exhibit similar trends, which align with the typical micro-arc oxidation (MAO) process for titanium and can be broadly divided into three stages. In the first stage, analogous to conventional anodic oxidation, the voltage rises rapidly within the initial 35–40 s, reaching approximately 400 V. During this period, a relatively dense initial oxide layer forms on the titanium surface. Gas release is observed on the sample surface prior to the onset of micro-arc discharges. In the second stage, around 40 s into the process, the slope of the voltage–time curve decreases, indicating the barrier effect of the initial oxide layer formed in the first stage. At this point, micro-arc discharges begin to appear and move across the sample surface. The initial oxide layer is broken through by these micro-discharges, allowing the voltage to continue rising steadily. During this stage, bright spots and slow-moving sparks become increasingly intense on the sample surface. The process is accompanied by unique plasma-chemical reactions, as well as repetitive breakdown, melting, and re-sintering of the coating. After approximately 110 s, the rate of voltage increase slows, marking the transition into the third stage. During this stage, bright and intense micro-arc discharges are observed, accompanied by loud cracking sounds, indicative of vigorous plasma activity on the sample surface. With increasing concentrations of Nb_2_O_5_ nanoparticles in the electrolyte, the overall voltage rises, particularly in the third stage, where the stable voltage increases from approximately 476 V at 0 g/L to about 490 V at 10 g/L. This suggests that the addition of Nb_2_O_5_ nanoparticles enhances the growth rate of the MAO film, which is consistent with other reports on the effects of nanoparticle additives. The observed effect can be attributed to the incorporation of Nb_2_O_5_ nanoparticles into the MAO film under the influence of the electric field and micro-arc discharges. Through plasma discharge sintering and melting processes, these nanoparticles become effectively doped into the film. Detailed characterization of the structural and compositional changes in the resultant films is presented in the following sections.

### 3.2. Characterization of MAO Thin Fims

**Figure 3:**
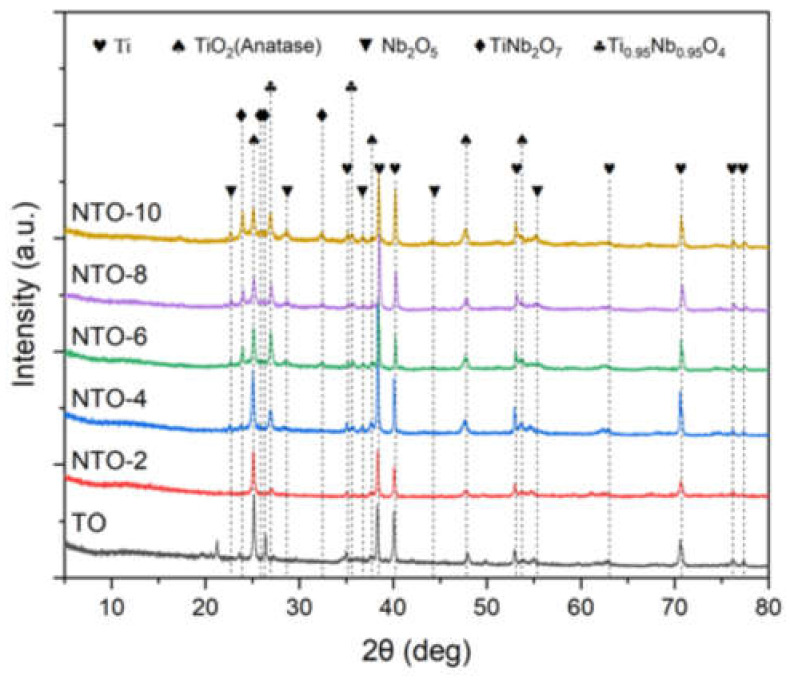
XRD patterns of TO, NTO-2, NTO-4, NTO-6, NTO-8, and NTO-10 samples.

Figure 3 reveals the phase composition changes in MAO coatings fabricated at different Nb_2_O_5_ concentrations. The TO sample surface is mainly composed of the α-Ti (JCPDS No. 44-1294) phase and the TiO_2_ (anatase, JCPDS No. 21-1272) phase. However, after doping with different concentrations of Nb_2_O_5_, three new phases were observed on the surfaces of NTO-2, NTO-4, NTO-6, and NTO-8 samples: Nb_2_O_5_ phase (JCPDS No. 30-0873), TiNb_2_O_7_ phase (JCPDS No. 28-1360), and Ti_0.95_Nb_0.95_O_4_ phase (JCPDS No. 30-0873). Diffraction peaks at 22.6°, 28.4°, 36.6°, and 55.1° exhibit a relatively large full width at half maximum (FWHM). The average crystalline size of Nb_2_O_5_ in the film is calculated by the Scherrer equation:

(2) D=Kλ/βcosθ

where K is the Scherrer constant (0.9), β is the FWHM of the diffraction peak, λ is the wavelength of Cu Kα X-ray (0.154 nm), and θ is the Bragg angle, which is half of the diffraction angle. The average crystalline size of Nb_2_O_5_ calculated from the Nb_2_O_5_ (130) plane is 24.5 nm, indicating that most of the incorporated Nb_2_O_5_ particles remain in the nanocrystalline structure. In addition to Nb_2_O_5_, two new Nb-doped TiO_2_ phases were observed in the Nb_2_O_5_-doped MAO samples, suggesting that the Nb_2_O_5_ doping process during MAO entails not only the incorporation and sintering of Nb_2_O_5_ nanoparticles, but also chemical reactions and the formation of new compounds under the influence of micro-arc discharge. The formation of TiNb_2_O_7_ and Ti_0.95_Nb_0.95_O_4_ phases during the MAO process is driven by high-temperature plasma micro-discharges and a strong electric field. During the plasma discharge and reaction, Nb_2_O_5_ nanoparticles are ionized in the electrolyte, releasing Nb^5+^; ions, which integrate into the TiO_2_ matrix through substitution and diffusion, ultimately leading to the nucleation of these mixed-metal oxides. The TiNb_2_O_7_ and Ti_0.95_Nb_0.95_O_4_ phases exhibit nanocrystalline structures with mixed-metal oxide characteristics, offering enhanced lattice distortions and improved functional properties, such as conductivity and catalytic activity [29,30].

Notably, with the increase in Nb_2_O_5_ concentration, the diffraction peak intensities of the three newly formed phases all show an increasing trend. The diffraction peak intensity of the TiO_2_ (anatase) phase at the 2-theta angle of 25.3° exhibits a decreasing trend, which may be due to the transformation of TiO_2_ (anatase) phase to other new phases or lattice distortion caused by the doping of Nb_2_O_5_. Meanwhile, the diffraction peak intensities of TiO_2_ (anatase) at other diffraction angles do not change much, indicating that this transformation or distortion may have a certain directionality. In summary, doping with different concentrations of Nb_2_O_5_ significantly affects the phase composition of MAO coatings, promotes the formation of new phases, and alters the diffraction peak intensities of the original phases. These findings are of great significance for understanding the impact of Nb_2_O_5_ doping on the properties of MAO coatings.

**Figure 4:**
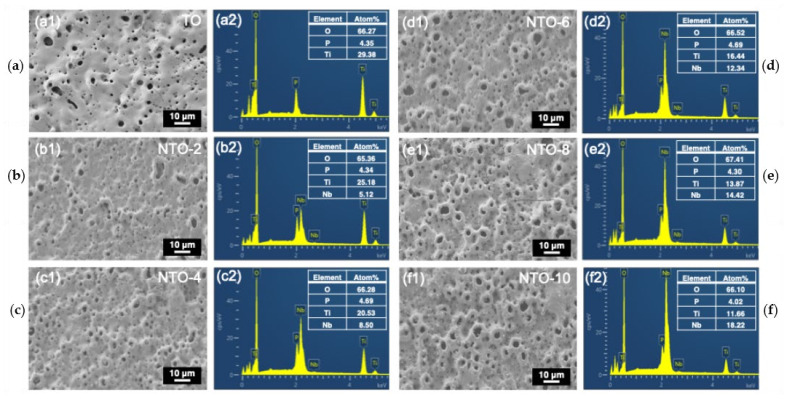
SEM images (**a1**,**b1**,**c1**,**d1**,**e1**,**f1**) and related EDS spectra (**a2**,**b2**,**c2**,**d2**,**e2**,**f2**) showing the surface morphology and elemental composition of different samples: (**a**) TO, (**b**) NTO-2, (**c**) NTO-4, (**d**) NTO-6, (**e**) NTO-8, and (**f**) NTO-10.

Figure 4 presents the SEM images and EDS spectra results of MAO coatings doped with varying concentrations of Nb_2_O_5_. All samples exhibit the characteristic surface morphology of MAO coatings, featuring a crater-like porous film structure formed by micro-arc discharges. With increasing Nb_2_O_5_ doping concentration, the pore size on the surfaces of NTO-2, NTO-4, NTO-6, NTO-8, and NTO-10 samples increases, which can be due to the progressively intensified discharge process with the increasing concentration of Nb_2_O_5_ nanoparticles. Notably, the NTO-4 sample demonstrates not only smaller pores but also a more uniform distribution. Furthermore, elemental enrichment analysis reveals no significant variation in the concentration of O and P elements across all samples, while the Ti content gradually decreases, and Nb content progressively increases. This observation aligns with the XRD results, confirming that the degree of Nb_2_O_5_ incorporation into the films is proportional to its concentration in the electrolyte.

**Figure 5:**
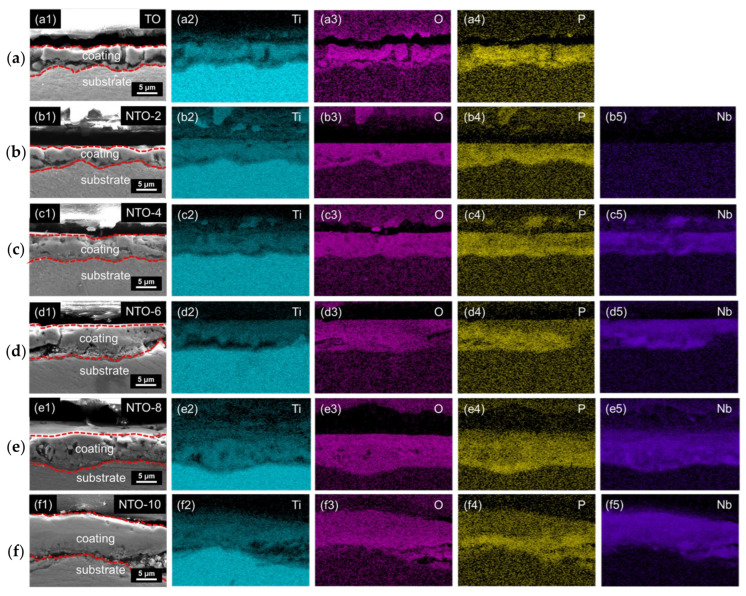
Cross-sectional SEM images (**a1**,**b1**,**c1**,**d1**,**e1**,**f1**) and related EDS mapping results (**a2–a4**,**b2–b5**,**c2–c5**,**d2–d5**,**e2–e5**,**f2–f5**) of different samples: (**a**) TO, (**b**) NTO-2, (**c**) NTO-4, (**d**) NTO-6, (**e**) NTO-8, and (**f**) NTO-10.

To further analyze the composition and structure of the coatings, the cross-sectional morphology and elemental mapping results of the samples after MAO treatment are shown in Figure 5. As seen in Figure 5(a1,b1,c1,d1,e1,f1), the coating thickness increases with the Nb_2_O_5_ concentration, with the average thickness of the films measured as 5.3, 4.8, 5.8, 7.3, 7.8, and 9.6 µm for TO through NTO-10 samples, respectively. The EDS mapping results (Figure 5(a2–a4,b2–b5,c2–c5,d2–d5,e2–e5,f2–f5)) indicate a uniform distribution of Nb within the films. During the micro-arc oxidation process, Nb_2_O_5_ nanoparticles in the weakly alkaline electrolyte are negatively charged and migrate toward and accumulate on the substrate surface under the influence of the electric field. These nanoparticles participate in plasma reactions and undergo repeated melting and sintering under the action of micro-arc discharges. Combined with the XRD results in Figure 3, it is evident that, in addition to reacting to form TiNb_2_O_7_ and Ti_0.95_Nb_0.95_O_4_, a significant amount of Nb_2_O_5_ incorporated into the coating remains in the form of nanoparticles, uniformly dispersed within the primary anatase TiO_2_ phase. These findings demonstrate that the MAO treatment of Ti in Nb_2_O_5_-doped electrolytes enables the fabrication of uniform NTO thin films.

**Figure 6:**
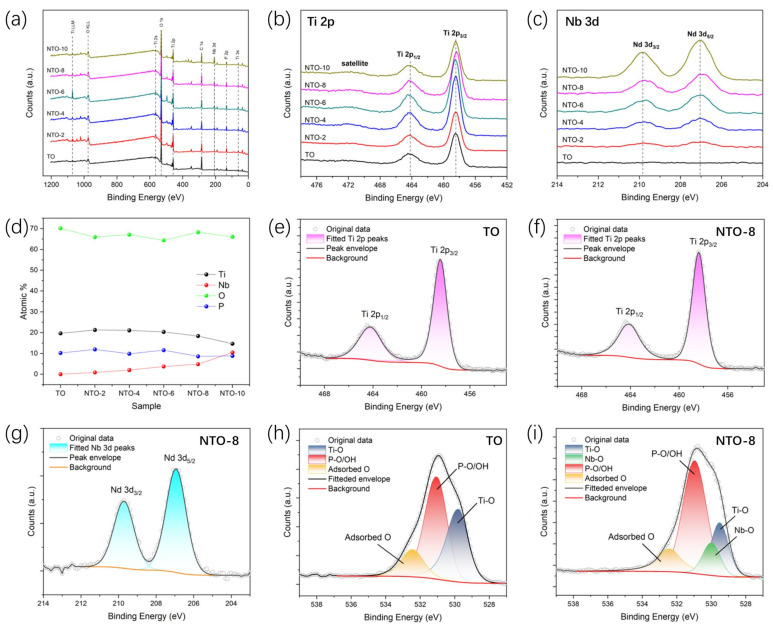
(**a**) XPS survey and high-resolution XPS spectra of Ti 2p (**b**) and Nb 3d (**c**) for different samples. (**d**) Atomic percentage of Ti, Nb, O, and P calculated from XPS spectra of different samples. Peaks deconvolution results of (**e**) Ti 2p for TO, (**f**) Ti 2p for NTO-8, (**g**) Nd 3d for NTO-8, (**h**) O 1s for TO, and (**i**) O 1s for NTO-8.

The surface oxidation state and chemical composition of the samples prepared from undoped and different concentrations of Nb_2_O_5_ electrolyte were determined using XPS. The binding energy of the XPS spectra measured for all the samples was calibrated with respect to the C1s peak position and set to 284.6 eV. Figure 6a shows the peak spectra corresponding to each element on the surface of the prepared samples under different working conditions. As shown in Figure 6b,c, the peaks of Ti 2p1/2 and Ti 2p3/2 are 464.3 eV and 458.5 eV, respectively, and the difference in the peaks between them is 5.8 eV. The peaks of Nb 2p3/2 and Nb 2p5/2 are 209.4 eV and 207.1 eV, respectively, and the difference in the peaks between them is 2.3 eV. Because of the large content of Ti in the samples, the peak spectra of Ti 2p3/2 and Nb 2p3/2 under different working conditions are not as high as the peak spectra of Nb 2p5/2. The Ti 2p peaks are in good agreement under different working conditions. However, due to the strong spin–orbit coupling effect of Nb^5+^ in Nb_2_O_5_, the intensity of the Nb 3d3/2 and Nb 3d5/2 splitting peaks gradually increases with the increas in the Nb_2_O_5_ concentration in the electrolyte. This result can indirectly indicate that the microarc oxidation technique can effectively dope niobium elements on the sample surface by increasing the number of Nb_2_O_5_ nanoparticles added to the electrolyte. Figure 6e–g shows the peak fits of elemental Ti on the surface of the TO sample, elemental Ti and elemental Nb on the surface of the NTO-8 sample in turn, and it can be seen that these two metal elemental states mainly exist in the low-valent and high-valent states, and the elemental states of Ti have different proportions under different working conditions. Figure 6h,i shows the peak fitting of each element on the surface of TO and NTO-8 samples; it can be clearly seen that Nb oxides appear on the surface of doped NTO-8 samples, and the percentage of Ti oxides decreases.

### 3.3. Hydrogen Sensing Tests of MAO Thin Films

**Figure 7:**
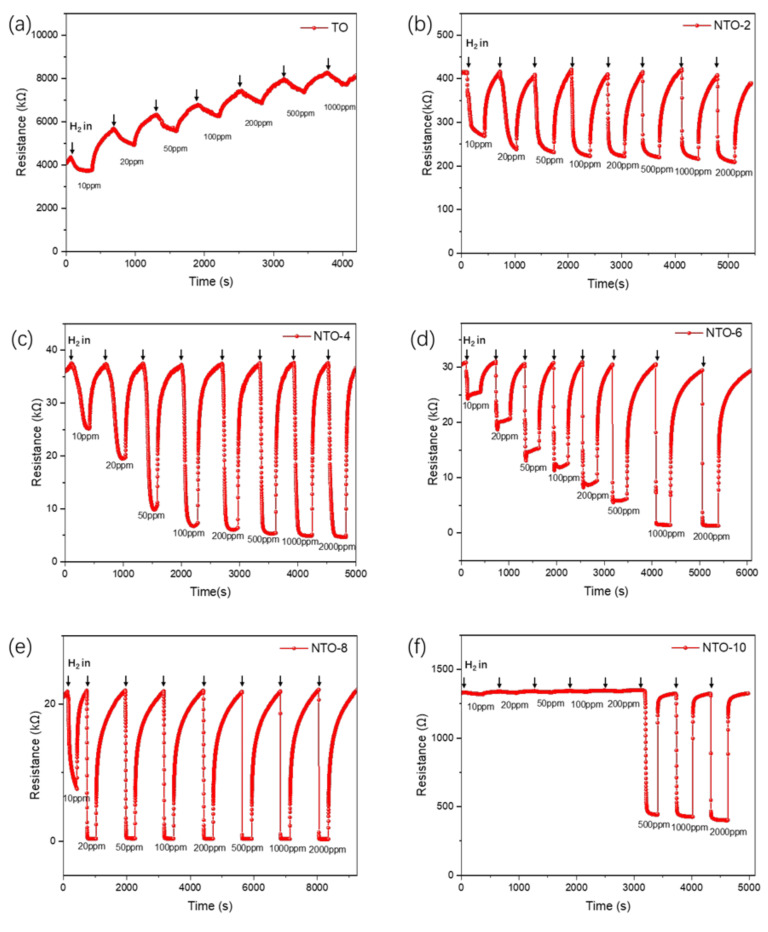
Dynamic response of the sensors based on (**a**) TO, (**b**) NTO-2, (**c**) NTO-4, (**d**) NTO-6, (**e**) NTO-8, and (**f**) NTO-10 toward 10, 20, 50, 100, 200, 500, 1000, and 2000 ppm hydrogen.

The hydrogen sensing behaviors of different samples are illustrated in Figure 7. During the hydrogen sensing tests, the TO sample exhibited significant baseline drift, making it difficult to calculate the response (*S*) despite resistance fluctuations upon exposure to H_2_; concentrations ranging from 10 ppm to 1000 ppm. In contrast, the NTO-2, NTO-4, NTO-6, and NTO-8 samples demonstrated a consistent and systematic increase in resistance when exposed to H_2_ concentrations of 10 ppm to 2000 ppm, indicating superior hydrogen sensitivity. The calculated *S* was used to evaluate hydrogen sensor performance. Among these samples, NTO-2 showed response values ranging from 35.3% to 49.7% across the tested concentration range. However, beyond 100 ppm (*S* = 46.3%), the increase in *S* became very slow, limiting its ability to accurately measure higher hydrogen concentrations. NTO-4 displayed a broader response range, with *S* increasing significantly from 32.7% to 80.4% at concentrations between 10 ppm and 100 ppm, though the increase diminished at higher concentrations. In comparison, NTO-6 exhibited a steady and continuous increase in resistance across the entire range, with response values ranging from 17.4% to 95.7%, demonstrating a wider detection range and higher stability. NTO-8 achieved exceptional sensitivity, with a response of 65.1% at 10 ppm, but increased to 98.4% at 20 ppm. Its response increase was minimal at higher concentrations, reaching 97.5% at 2000 ppm. Similarly, NTO-10 maintained a high response value of over 95% throughout the range of 10 ppm to 2000 ppm, but its sensitivity to changes in concentration was limited. Based on these findings, further analysis and discussion focused on the sensing properties of the NTO-2, NTO-4, and NTO-6 samples to evaluate their performance and applicability in hydrogen sensing.

**Figure 8:**
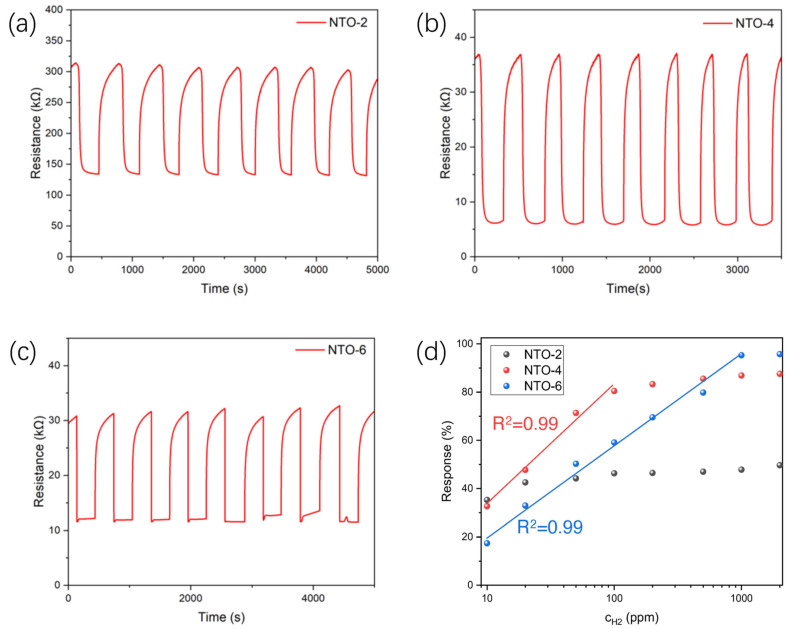

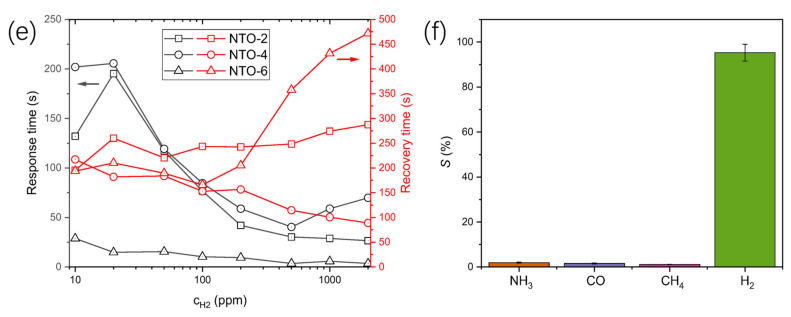
(**a**–**c**) Repeatability of (**a**) NTO-2, (**b**) NTO-4, and (**c**) NTO-6 to 100 ppm H_2_ at room temperature. (**d**) Relationship between response (*S*) and H_2_ concentration (*C_H2_*) for NTO-2, NTO-4, and NTO-6, with lines showing linear relationship between *S and lgC_H2_*. (**e**) Response time and recovery time for NTO-2, NTO-4, and NTO-6 to 10-2000 ppm H_2_. (**f**) Depiction of the selectivity of NTO-6 to 1000 ppm NH_3_, CO, CH_4_, and H_2_.

Figure 8a–c show the resistance responses of the NTO-2, NTO-4, and NTO-6 samples under repeated cycles of exposure to 100 ppm H_2_ and air. It can be observed that all three sensors exhibit excellent responses of repeatability and reversibility. The responses of the NTO-2, NTO-4, and NTO-6 samples as a function of hydrogen concentration are shown in Figure 8d. It can be observed that the NTO-4 and NTO-6 samples exhibit a linear relationship with the logarithmic value of hydrogen concentration (lg*C_H_2__*) within certain ranges, specifically 10–100 ppm for NTO-4 and 10–1000 ppm for NTO-6. Notably, the NTO-6 sample shows a significant increase in S within its linear range, which is critical for accurately detecting hydrogen concentrations. Figure 8e presents the response time (*t_res_*) and recovery time (*t_rec_*) of different sensors as a function of hydrogen concentration. As the hydrogen concentration increases, the response time of the sensors generally decreases, owing to the faster reaction rate at higher H_2_ concentrations. Among the samples, NTO-6 exhibits the shortest response time overall, with a response time of just 28.7 s at 10 ppm and approximately 3.5 s at 2000 ppm. Regarding recovery time, NTO-4 performs better overall, displaying shorter recovery times compared to NTO-2 and NTO-6, with recovery time decreasing further as the hydrogen concentration increases. As seen in Figure 8f, the selectivity of the sensors was assessed by testing the NTO-6 sample with H_2_ and interference gasses such as NH_3_, CH_4_, and CO. The results show that NTO-6 exhibits superior resistance to interference from these gasses, highlighting its strong selectivity for hydrogen detection.

## 4. Discussion

The resistance-based sensing mechanism of semiconductor metal oxides (SMOs) is intricate and has been extensively studied [5,31,32]. The widely accepted explanation revolves around changes in the surface electron depletion region caused by interactions between hydrogen and chemisorbed oxygen species on the surface. In n-type SMOs like SnO_2_, TiO_2_, and ZnO, oxygen molecules in the air absorb onto the surface, forming physisorbed or chemisorbed oxygen species (O_2_^−^, O^−^, and O^2−^). These oxygen species capture electrons from the conduction band of the SMOs, effectively reducing the free electron density in the material. This process creates an electron depletion region near the surface, significantly increasing the resistance by reducing the net carrier density. When exposed to hydrogen, the adsorbed oxygen species (O_2_^−^, O^−^, or O^2−^) on the sensor surface react with hydrogen to form water (H_2_O), releasing trapped electrons back into the conduction band. This reduces the depletion layer width and lowers the potential barrier energy (Φ_B_), thereby decreasing resistance. This mechanism aligns with the ionosorption model of hydrogen sensing [5].

Based on the characterization results from XRD, XPS, SEM, and EDS, the NTO thin films formed by the MAO show a homogeneous dispersion of Nb_2_O_5_ within the TiO_2_ matrix, with the Nb:Ti ratio increasing with the Nb_2_O_5_ concentration in the electrolyte. During the MAO process, most of the Nb_2_O_5_ retains its crystalline structure, while a portion undergoes transformation through plasma reactions induced by micro-arc discharges. Since both Nb_2_O_5_ and anatase TiO_2_ are n-type semiconducting metal oxides, when Nb_2_O_5_ and TiO_2_ are exposed to hydrogen, adsorbed oxygen molecules $O_{2}(ads)$ capture electrons and convert into oxygen species $O_{2}^{-}$, reducing the electron concentration in the TiO_2_ base, which leads to an increase in resistance. Consequently, an electron depletion layer (EDL) and potential barrier energy ${ɸ}_{B}$ are formed [33]. The reactions in the air are displayed as follows:

(3) $$O_{2}\left(gas\right)↔O_{2}\left(ads\right)$$

(4) $$O_{2}\left(ads\right)+e^{-}↔O_{2}^{-}$$

(5) $$O_{2}^{-}\left(ads\right)+e^{-}↔2O^{-}\left(ads\right)$$

Hydrogen molecules can chemisorb on the surface of TiO_2_ in a hydrogen atmosphere, react with the oxygen species and form H_2_O. This reaction releases trapped electrons back into the TiO_2_. During this process, the EDL and potential barrier energy ${ɸ}_{B}$ decrease, resulting in lower resistance. The reaction is as shown below:

(6) $$H_{2}+O^{-}\left(ads\right)\to H_{2}O$$

The depletion layer model provides a clear explanation of the gas sensing mechanism in the TiO_2_/Nb_2_O_5_ system. TiO_2_ has an electron affinity (χ) of 3.56 eV, whereas Nb_2_O_5_ possesses a higher electron affinity. Additionally, TiO_2_ features a wider energy gap (E_g_ = 3.4 eV) compared to Nb_2_O_5_ (E_g_ = 3.2 eV). Generally, the larger bandgap of TiO_2_ gives it a higher Fermi level, classifying it as a semiconductor or insulator. In contrast, Nb_2_O_5_, with its lower Fermi level, exhibits more metallic or semi-metallic characteristics. When TiO_2_ and Nb_2_O_5_ are in contact, electrons flow from the higher Fermi level of TiO_2_ to the lower Fermi level of Nb_2_O_5_ until the equilibrium is reached, causing conduction band bending to equalize energy levels across the interface.

When Nb is doped into TiO_2_, the work function differences (4.25 eV for Nb_2_O_5_ and 3.87 eV for TiO_2_) drive electron transfer, rearranging the Fermi levels to establish thermal equilibrium in the heterostructure. The spillover effect further enhances the performance of Nb-doped TiO_2_ by enabling the doped surface to capture a greater number of reactive oxygen species O^−^ [34,35]. The increased barrier height in the depletion layer results in a higher response when the sensors encounter hydrogen [18].

The Nb/Ti composite metal oxides in NTO thin film, such as NbTi_2_O_7_ and Ti_0.95_Nb_0.95_O_4_, also play a significant role in enhancing the overall performance of NTO thin film. During the preparation of the NTO thin films, the plasma-induced high-temperature reactions during micro-arc discharges facilitate the formation of doped oxides such as NbTi_2_O_7_ and Ti_0.95_Nb_0.95_O_4_ within the film. Meanwhile, the film retains anatase-phase TiO_2_ and partially reacts to Nb_2_O_5_ nanoparticles, collectively forming the NTO film. The XRD results reveal that the content of these oxides increases with the concentration of Nb_2_O_5_ nanoparticles in the electrolyte. As a result, the initial resistance (R_a_) of the NTO samples during hydrogen sensing tests is significantly lower compared to the TO sample, progressively decreasing from NTO-2 to NTO-10. These compounds of NbTi_2_O_7_ and Ti_0.95_Nb_0.95_O_4_ likely contribute to enhanced electron mobility and additional adsorption sites [30]. Their unique crystal structure may facilitate better charge carrier movement compared to pure TiO_2_ [36]. The mixed-phase interface may offer more active sites for oxygen and hydrogen adsorption, improving sensitivity. These compounds might also exhibit catalytic effects, promoting faster reactions between hydrogen and adsorbed oxygen species, further enhancing sensor performance.

## 5. Conclusions

This study demonstrates the successful one-step synthesis of Nb-doped TiO_2_ (NTO) thin films via the MAO technique for semiconductor-based hydrogen sensors. The incorporation of Nb_2_O_5_ nanoparticles during the MAO process led to the formation of mixed oxides, such as NbTi_2_O_7_ and Ti_0.95_Nb_0.95_O_4_, alongside anatase-phase TiO_2_, resulting in a composite film with enhanced electrical conductivity and hydrogen sensing performance. The as-prepared NTO-2, NTO-4, and NTO-6 samples possess good sensitivity to different concentrations of hydrogen at room temperature. Among these samples, NTO-6 demonstrated the most promising hydrogen sensor performance, showing a broad detection range (10–2000 ppm), excellent sensitivity (17.4–95.7%), and fast response times (<3 s). The strong linear relationship between response values and hydrogen concentration for NTO-6 further underscores its potential for precise and reliable hydrogen detection. The enhanced hydrogen sensing mechanism of NTO thin films primarily stems from the influence of Nb_2_O_5_ nanoparticle doping in the anatase-phase TiO_2_ structure on the semiconductor surface depletion layer. Additionally, the Nb/Ti composite metal oxides, such as NbTi_2_O_7_ and Ti_0.95_Nb_0.95_O_4_, improve charge transfer and provide additional adsorption sites. In summary, the Nb-doped TiO_2_ thin films prepared through MAO not only exhibit high hydrogen sensitivity at room temperature but also address challenges associated with TiO_2_-based hydrogen sensors, such as limited response and high operating temperatures. These results provide valuable insights into the design and optimization of TiO_2_-based hydrogen sensors, paving the way for further advancements in gas-sensing technologies for clean energy applications.

## Data Availability

The data that support the findings of this study are available upon reasonable request from the authors.
